# Kinetic transcriptome analysis reveals an essentially intact induction system in a cellulase hyper-producer *Trichoderma reesei* strain

**DOI:** 10.1186/s13068-014-0173-z

**Published:** 2014-12-12

**Authors:** Dante Poggi-Parodi, Frédérique Bidard, Aurélie Pirayre, Thomas Portnoy, Corinne Blugeon, Bernhard Seiboth, Christian P Kubicek, Stéphane Le Crom, Antoine Margeot

**Affiliations:** IFP Energies nouvelles, 1-4 avenue de Bois-Préau, 92852 Rueil-Malmaison, France; Sorbonne Universités, UPMC Univ Paris 06, Institut de Biologie Paris-Seine (IBPS), F-75005 Paris, France; Research Division Biotechnology and Microbiology, Institute of Chemical Engineering, Technische Universität Wien, Getreidemarkt 9/166, A- 1060 Vienna, Austria; Austrian Center of Industrial Biotechnology, 8010 Graz, Austria; Ecole Normale Supérieure, Institut de Biologie de l’ENS, IBENS, Plateforme Génomique, Paris, F-75005 France; Ecole Normale Supérieure, Institut de Biologie de l’ENS, IBENS, Inserm, U1024, Paris, F-75005 France; Ecole Normale Supérieure, Institut de Biologie de l’ENS, IBENS, CNRS, UMR 8197, Paris, F-75005 France

**Keywords:** Biofuels, Cellulase, Fed-batch, *Trichoderma reesei*, Transcriptome, Systems biology

## Abstract

**Background:**

The filamentous fungus *Trichoderma reesei* is the main industrial cellulolytic enzyme producer. Several strains have been developed in the past using random mutagenesis, and despite impressive performance enhancements, the pressure for low-cost cellulases has stimulated continuous research in the field. In this context, comparative study of the lower and higher producer strains obtained through random mutagenesis using systems biology tools (genome and transcriptome sequencing) can shed light on the mechanisms of cellulase production and help identify genes linked to performance. Previously, our group published comparative genome sequencing of the lower and higher producer strains NG 14 and RUT C30. In this follow-up work, we examine how these mutations affect phenotype as regards the transcriptome and cultivation behaviour.

**Results:**

We performed kinetic transcriptome analysis of the NG 14 and RUT C30 strains of early enzyme production induced by lactose using bioreactor cultivations close to an industrial cultivation regime. RUT C30 exhibited both earlier onset of protein production (3 h) and higher steady-state productivity. A rather small number of genes compared to previous studies were regulated (568), most of them being specific to the NG 14 strain (319). Clustering analysis highlighted similar behaviour for some functional categories and allowed us to distinguish between induction-related genes and productivity-related genes. Cross-comparison of our transcriptome data with previously identified mutations revealed that most genes from our dataset have not been mutated. Interestingly, the few mutated genes belong to the same clusters, suggesting that these clusters contain genes playing a role in strain performance.

**Conclusions:**

This is the first kinetic analysis of a transcriptomic study carried out under conditions approaching industrial ones with two related strains of *T. reesei* showing distinctive cultivation behaviour. Our study sheds some light on some of the events occurring in these strains following induction by lactose. The fact that few regulated genes have been affected by mutagenesis suggests that the induction mechanism is essentially intact compared to that for the wild-type isolate QM6a and might be engineered for further improvement of *T. reesei.* Genes from two specific clusters might be potential targets for such genetic engineering.

**Electronic supplementary material:**

The online version of this article (doi:10.1186/s13068-014-0173-z) contains supplementary material, which is available to authorized users.

## Background

Random mutagenesis followed by strain screening, using either chemical substances or irradiation, has for decades been the choice method for developing efficient microbial industrial strains. The main drawbacks of random mutagenesis are well known: accumulation of deleterious mutations leading to evolutionary dead ends or unstable strains, limited or nonexistent ability to select for synergistic mutations, due to the low probability that the two or more suitable mutations appear in a single clone, and finally few or no clues regarding the underlying cellular mechanisms involved [1]. Nevertheless, this empirical approach has been very successful and remains broadly used even more than 25 years after the advent of recombination technologies. A spectacular example is the filamentous fungus *Trichoderma reesei*, famous for its high cellulase enzymes secretion capacity, which is of high interest for the second generation biofuel industry [2]. Industrial strains derived from the original QM6a strain isolated on the canvas tents of US soldiers are able to produce 40 to 100 g L^-1^ cellulase enzymes, which represent a more than 1,000-fold production improvement [3]. *T. reesei* is the workhorse of cellulase production, and these enzymes are essential for the economic feasibility of biorefinery processes that rely on fermentation of lignocellulosic biomass sugar monomers. However, despite those high production titres, the specific productivity (g/g.h^-1^) of the strains remains relatively low and there is still a need for strains with higher productivities.

Earlier studies on cellulase production regulation have identified the XYR1 transcription factor as a pivotal inducer of cellulase production [4-6] and have also assessed a role for the carbon catabolite repression mediated by the CRE1 transcription factor [7-9]. Indeed, strains deleted for CRE1 function show higher production levels [10]. Moreover, the RUT C30 strain, one of the most studied hyper-producing strains, bears a truncated version of the *cre1* gene that has been demonstrated to be inactive and to allow higher cellulase production [11]. The presence of inducers in extracellular media (cellulose, hemicellulose, cellobiose, but also lactose and sophorose) leads to *xyr1* transcription activation through a yet-to-be-discovered transduction mechanism [12].

Other transcription factors and genes involved in regulation have been identified, but their respective roles remain enigmatic (*ace1, ace2, ace3, lae1, creD*) [13-17]. The importance of a low growth rate to reach a good cellulase productivity was also pointed out [18]. Indeed, most industrial protocols are based on a fed-batch of the inducing carbon source, leading to cellulase production and practically zero cellular biomass production [19].

The *T. reesei* genome was sequenced in 2005 [20], followed by sequencing of high and low producer strains by various groups [21-25]. These studies uncovered many mutations, some of which affected genes that could reasonably be associated with cellulase production [21]. Indeed, other studies led to formal linking of some of these genes with a higher cellulase production phenotype [17,23]. However, the majority of mutations have not been characterized, owing to the somewhat labour-intensive genetics of *Trichoderma*. Moreover, random mutagenesis probably led to silent or unfavourable mutations, making systematic assaying of each mutation an unattractive strategy.

A way to further select target genes before switching to labourious genetic validations is to perform transcriptome or other systems biology analysis in conditions approaching industrial ones. A simple assumption would be that a significant number of mutated genes will be transcribed, induced or repressed under these conditions, helping to define or refine the studied system further. Microarrays give a reliable picture of induced or repressed genes under given conditions, and RNA sequencing is even more powerful, as it allows more sensitive and quantitative detection of a given transcript, even if no expression variation is observed. Indeed some transcriptome studies have been performed on *T. reesei* [17,26-28], and some have led to the identification of genes directly linked to cellulase production. Most of these studies have been performed on the relatively low producer strain QM9414 [26-28] and rely on batch cultures, with the exception of [17], which has used chemostat culture and the RUT C30 strain.

Despite identification of genes having an effect on cellulase production, the performance of historical strains obtained through random mutagenesis is still not matched by strains modified solely by targeted genetic engineering. The reason is probably that the “roots of cellulase production” have still not been properly understood.

In this study, a transcriptome analysis was performed to identify genes involved specifically in protein production by *T. reesei* under conditions close to those of an industrial process (bioreactor, monitored pH, soluble and cheap inducing carbon source like lactose in fed-batch). In our opinion, these would be the most probable candidates to have a genuine effect on productivity in an industrial context when modified. We also wanted to decipher the chain of events of early induction; therefore, a kinetic study was performed over the first 24 h after the start of the induction. We chose to work with the respectively moderate and higher producer strains NG 14 and RUT C30, which our group previously sequenced [21]. This allowed us to perform a close comparison of transcriptomes and genomes from these two strains and from other lineages.

We show that a smaller number of genes than previously reported are involved in cellulase production, probably owing to well-controlled culture conditions. Moreover, the complexity of the kinetic pattern observed and the differences between the two strains suggest that inactivation of catabolic repression through *cre1* deletion may not be the only important genetic event that happens during the breeding of RUT C30. We also show that only a small number of genes that have been mutated during the obtention of these strains are differentially expressed by the transcriptome analysis and that they have the same regulation patterns.

## Results

### NG 14 and RUT C30 strains’ protein production in an industrial cultivation regime

In order to study the early phase of induction by lactose of *T. reesei,* we cultivated in duplicate strains RUT C30 and NG 14 in a bioreactor by following a previously described protocol that mimics an industrial process [19,29]. Each strain was grown on D-glucose during the batch phase for around 24 h, until the glucose was depleted. The glucose concentration decline was monitored, and lactose fed-batch cultivation was started when equal to or below 0.5 g L^-1^. This lactose pulse is tuned in such a way that no lactose accumulates in the medium, which favours cellulolytic enzymes production, based on previous experience ([29,30]). This induction step is called the fed-batch phase. Cultivations were carried out up to 48 h after fed-batch start, except for one NG 14 replicate that had to be stopped shortly after 24 h because of feeding pump failure and one for RUT C30 that was conducted up to 148 h.

**Figure 1:**
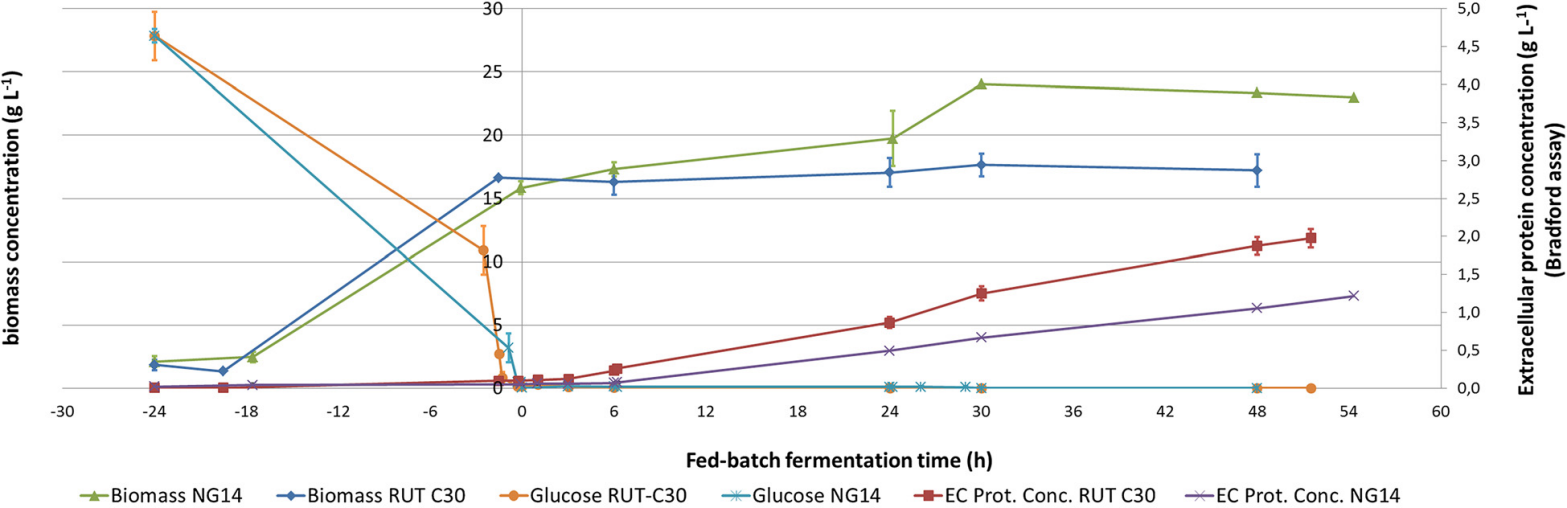
**RUT C30 and NG 14 bioreactor culture profiles.** Growth curves (dry biomass concentration g.L^-1^) and protein production levels (extracellular protein concentration g.L^-1^ (EC Prot. Conc.), as assayed by Bradford method (see Results), are displayed for NG 14 and RUT C30 *T. reesei* strains. Time 0 h marks the start of lactose feeding. Negative values represent the 24 h of batch culture and positive values the first 48 h of fed-batch culture with lactose. Each curve represents the average measure of two independent cultures; error bars show average standard deviation to give an estimate of replicates’ quality (excluding technical replicates). One NG 14 replicate is lacking after 24 h lactose induction, so dispersion data is not available. Bradford method allows accurate assessment of production start and comparison between strains but underestimates actual values and is therefore not appropriate for carbon balancing (see Results for detailed explanations).

The cultivation data for each fermentation are presented in (Additional file 1: Figure S1). Carbon source concentrations and biomass and protein production, focussed on the first 48 h after fed-batch are presented in Figure 1, with merged cultivation duplicates. CO_2_ production was monitored to achieve carbon balances, which reached more than 0.90 gC_produced_/gC_consumed_ (data not shown). We chose to determine protein concentration using the Bradford method [31] to avoid background protein with peptides contained in the corn steep in the culture medium when protein concentration is low. However, for cellulases, this method is known to underestimate actual protein concentrations 3.5 to 5-fold [32] compared to the Lowry method [33], which reflects actual production. This conversion factor was used for mass balancing and to compare specific productivities with previous work (Jourdier *et al.* [29,30]). In addition, Lowry protein measurements were made on the RUT C30 cultivation that lasted for 150 h to check the consistency of the calculation (Additional file 2: Figure S2).

The batch phase resulted in a rapid consumption of glucose in less than 24 h, in a similar fashion for all fermentations (Figure 1). At the end of this phase, the average biomass of both the RUT C30 and NG 14 strains increased up to 16 g L^-1^. Lactose injection was started when the glucose concentration was below 1 g L^-1^. For all cultivations, no galactose or lactose was detectable in subsequent measurements during the fed-batch phase. The glucose concentration remained between 0.2 and 0 g L^-1^ (Additional file 3: Table S5).

During fed-batch, the biomass concentration of the RUT C30 and NG 14 strains remained fairly constant. A difference is seen at 48 h with 24 g L^-1^ for NG 14 and 17 g L^-1^ for RUT C30. However, the measure for NG 14 is based on only one replicate, as the second culture for this strain had to be stopped at 24 h, and we cannot conclude that the observed difference has any significance.

Protein production starts sharply as early as 3 h after lactose induction in the RUT C30 strain, with a steady production rate. The protein production starts between 6 and 24 h after induction in NG 14. As the production rate remains steady, it suggests that protein production indeed started closer to 6 h than to 24 h. The overall specific productivities of the strains between 4 h and 30 h were on average 1.2 mg/g_cell_/h and 3.98 mg/g_cell_/h for NG 14 and RUT C30, respectively. Applying a Bradford to Folin correction factor between 3.5 and 5 leads to actual estimated productivities between 4.2 and 6.0 mg/g_cell_/h for NG 14 and between 13.9 and 19.9 mg/g_cell_/h for RUT C30. Accordingly, the protein concentration at the end of production (148 h) reached 32.5 g/L for one of the RUT C30 strains, making a specific productivity of 13.4 mg/g_cell_/h. All these date are in line with the results of previously published work (up to 15 mg/g_cell_/h) [29].

Filter paper activities were measured and show values in line with data from previous work [19] and are homogeneous between strains and time points, suggesting no dramatic differences in cocktail compositions (data not shown).

### Global transcriptome changes during protein production

We sampled the fungal biomass to extract the total RNA after lactose addition to the medium. We wanted a focus on early induction and therefore chose 1, 3, 6 h after fed-batch. The choice of the final 24 h time points was based on our previous study, as a very high induction of cellulase genes was observed, suggesting that early induction events are over at this time [34]. We also used one sample point just before lactose induction as a reference (time 0) for each culture. This sampling process was carried out for the four independent bioreactor cultures, and the total RNA for each time point was hybridized against the time 0 reference on a transcriptome custom microarray designed for *T. reesei*. From a total of four hybridizations for each kinetic point, the microarray results were normalized and a differential analysis was performed.

**Figure 2:**
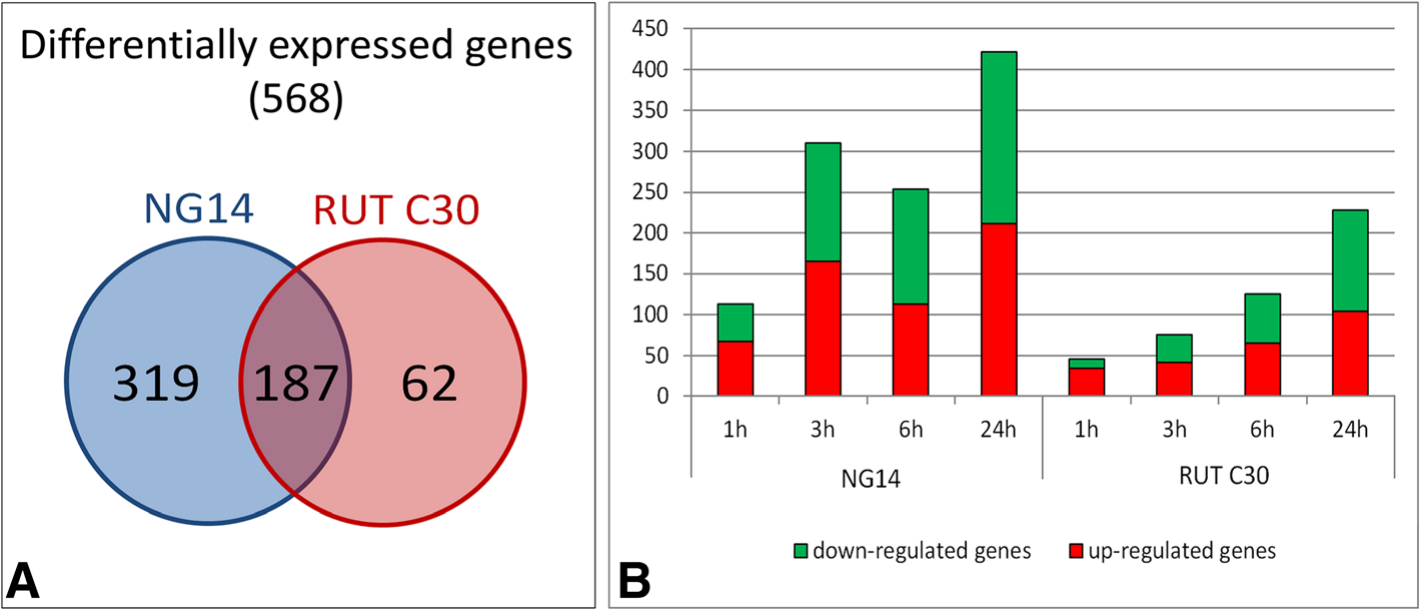
**Differentially expressed genes of NG 14 and RUT C30 during lactose induction. (A)** Venn diagram indicates the number of genes specific to each strain and the overlap between them. **(B)** The number of induced or repressed genes during induction by lactose is depicted in a bar chart. It displays the number of induced (red) and repressed (green) genes for the four time points (1, 3, 6 and 24 h) found during the induction in the RUT C30 and NG 14 strains compared to time 0. The differentially expressed genes have been selected using a 5% false discovery rate cut-off and with an absolute log2 fold change greater than 1.

After consolidation of duplicates and statistical analysis, we obtained 568 genes that were differentially expressed compared to their corresponding time 0, both strains considered. The differentially expressed genes specific to each strain are indicated in Additional file 4: Table S1. The numbers of differentially expressed genes found specifically for each strain and shared in both strains are shown in Figure 2A. Interestingly, RUT C30 showed five times fewer differentially expressed genes than NG 14 and only 62 specific genes.

The number of differentially expressed genes for each time point in each strain is shown in Figure 2B. We observed that for both strains the number of induced and repressed genes increases during induction, from 113 genes 1 h after induction to 422 after 24 h for NG 14. For RUT C30, the increase is from 46 genes 1 h after induction to 228 after 24 h.

The most striking difference between the strains is observed in the first hours of induction (between 1 and 3 h of induction). At those times, there is an increase in the number of induced genes in NG 14 from 67 to 165, while in RUT C30 this number remains about the same, suggesting that no significant expression changes occur in the early steps of induction in RUT C30. The increase in the number of induced genes in RUT C30 correlates with the start of active protein secretion 3 h after lactose feeding onset. More surprisingly, the number of repressed genes is quite high for the early induction stage for NG 14, being 145 at 3 h, 141 at 6 h and 211 at 24 h. In RUT C30, the repressed genes increase especially at late induction stages, with 60 genes at 6 h and 124 genes at 24 h.

### Clustering analysis reveals kinetic differences between NG 14 and RUT C30

**Figure 3:**
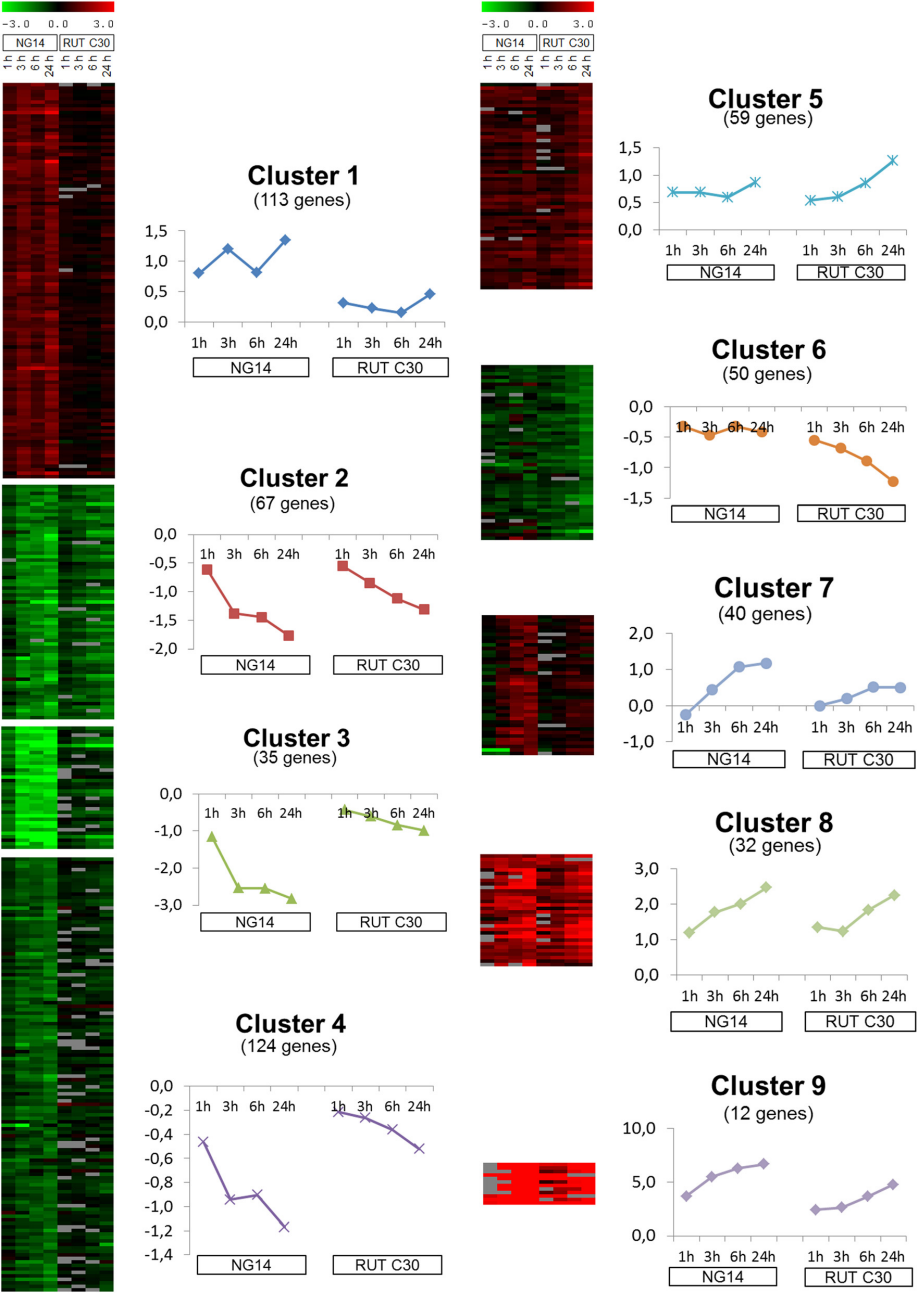
**Clustering of differentially expressed genes during induction by lactose in both studied strains.** From 568 genes identified as significantly regulated among the several expression experiments performed, 532 were gathered in 9 clusters according to their changes during the NG 14 and RUT C30 lactose induction. The average profile of each cluster is shown next to its heat map.

We applied clustering techniques to classify gene expression profiles into groups of similar patterns. From the expression matrix containing 568 differentially expressed genes, we were able to sort 532 of them into 9 clusters according to their expression pattern (Figure 3). All clusters can be classified as “up-regulated” (1, 5, 7–9) or “down-regulated” (2, 3, 4 and 6). Up-regulated clusters include 256 genes (45.1% of the matrix) and down-regulated gene clusters include 276 genes (48.6%).

Qualitative differences were observed in genes from up-regulated clusters between NG 14 and RUT C30. Cluster 7 group genes up-regulated at 6 h, only for NG 14. Conversely, cluster 5 group genes up-regulated later at 24 h, only for RUT C30. Group 1 is the largest cluster and shows early induction (1 h) for NG 14 with no changes in the RUT C30 strain. Clusters 8 and 9 are early up-regulated profiles that group genes showing up-regulation as early as 1 h after lactose induction for both strains. However, cluster 9 shows higher expression ratios than cluster 8, especially for the NG 14 strain.

Regarding the down-regulated genes, we saw marked differences in the kinetics between NG 14 and RUT C30. Most of these genes in NG 14 have an early repression pattern, at 1 h (cluster 3), and at 3 h (clusters 2 and 4) after lactose induction. Most down-regulated genes in the RUT C30 strain are found in cluster 2, with much lower expression ratios and retarded compared to NG 14. In contrast, cluster 6 gathers the only genes that are down-regulated in RUT C30 and with no noticeable expression variation in NG 14.

Only clusters 5 and 6 featured genes with an increase or decrease in gene expression specific to the RUT C30 strain, with no changes observed in the NG 14 strain. These clusters contain 59 of the 62 genes found specific to RUT C30 (Figure 2A). All these differential expressions take place at later times, when production has already started, and they may be pivotal in explaining the productivity differences observed. For the rest of the clusters, the differences between the two strains consist in up- or down-regulations that occur only in NG 14 or are severely reduced in RUT C30 (except for cluster 8, where the two strains share the same expression). Several hypotheses can be drawn from these results: regulation could have already occurred in RUT C30 before the lactose induction, it could be constitutive in RUT C30, or it could reflect genuine de-regulation regarding the lactose induction signal.

### Cluster characterization using functional categories

From a manual annotation of each up- or down-regulated *T. reesei* gene (see Materials and methods), genes were categorized according to the Functional Catalogue (FunCat) (Additional file 5: Table S2). The most representative categories (categories with more than ten genes in the whole dataset) and the number of genes found in each category in each cluster are shown in Table 1. Genes related to amino acid metabolism and ribosome biogenesis were found more in cluster 1, up-regulated only in NG 14. Genes linked to metabolism, lipid metabolism, energy metabolism and extracellular protein degradation were mostly found in clusters 2, 3 and 4. Cluster 2 contains the majority of lipid metabolism genes, while most genes for metabolism, energy metabolism and extracellular protein degradation were found in cluster 4. In addition, four proteasome components, *pre2*, *pre5*, *pre6* and *pre9* (Trire2:121009, Trire2:121343, Trire2:76010, Trire2:124031, respectively), were found in cluster 4, and the gene phosphoenolpyruvate carboxykinase *pck1* (Trire2:124115), key in gluconeogenesis, was found in cluster 3.

**Table 1:** **Main functional categories from transcriptome analysis in NG 14 and RUT C30 strains**

**Functional category**	**FunCat number**	**Cluster 1**	**Cluster 2**	**Cluster 3**	**Cluster 4**	**Cluster 5**	**Cluster 6**	**Cluster 7**	**Cluster 8**	**Cluster 9**	**Total category**
Ribosome biogenesis	12.01	**52** (96%)			1 (2%)		1 (2%)				**54**
Metabolism	01.00	3 (7%)	4 (9%)	3 (7%)	**22** (48%)	1 (2%)	7 (15%)	4 (9%)	2 (4%)		**46**
Extracellular metabolism (CAZymes)	01.25	1 (2%)	2 (4%)	3 (7%)	5 (11%)	5 (11%)	7 (15%)	2 (4%)	9 (20%)	**12** (26%)	**46**
Transported compounds	20.01	4 (9%)	6 (14%)	(7%)	8 (19%)	4 (9%)	1 (2%)	**10** (23%)	7 (16%)		**43**
Amino acid metabolism	01.01	**10** (24%)	7 (17%)	6 (14%)	7 (17%)	7 (17%)	3 (7%)	1 (2%)	1 (2%)		**42**
Lipid, fatty acid and isoprenoid metabolism	01.06	8 (22%)	**13** (36%)	2 (6%)	9 (25%)		1 (3%)	1 (3%)	2 (6%)		**36**
Transcriptional control	11.2.3.4	2 (10%)		1 (5%)	5 (25%)	**7** (35%)		4 (20%)	1 (5%)		**20**
Extracellular protein degradation	01.25.03	1 (6%)	4 (24%)	2 (12%)	**8** (47%)		2 (12%)				**17**
Energy metabolism	02.00	3 (19%)	4 (25%)	1 (6%)	**7** (44%)				1 (6%)		**16**
Stress response	32.01	1 (7%)	1 (7%)		4 (29%)		3 (21%)	5 (36%)			**14**
Vesicular transport (secretion)	20.09.07	1 (8%)	1 (8%)		1 (8%)	**7** (54%)			3 (23%)		**13**
Cellular communication, signal transduction	30.00	3 (23%)	2 (15%)		2 (15%)	4 (31%)	1 (8%)	1 (8%)			**13**
Secondary metabolism	01.20	2 (17%)	1 (8%)	2 (17%)	3 (25%)	1 (8%)	1 (8%)	2 (17%)			**12**
Detoxification	32.07	1 (9%)	3 (27%)	2 (18%)	4 (36%)		1 (9%)				**11**
Other categories		**10** (26%)	4 (10%)	4 (10%)	7 (18%)	5 (13%)	3 (8%)	4 (10%)	2 (5%)		**39**
Unclassified		4 (13%)	1 (3%)	5 (16%)	**13** (42%)	2 (6%)	5 (16%)		1 (3%)		**31**
Unknown protein		7 (9%)	14 (18%)	1 (1%)	**18** (23%)	16 (20%)	14 (18%)	6 (8%)	3 (4%)		**79**
**Total**		**113**	**67**	**35**	**124**	**59**	**50**	**40**	**32**	**12**	**532**

Only the FunCat categories with more than ten genes in the whole dataset are considered main functional categories. The number of genes belonging to categories is provided for each cluster. Category-associated genes found more abundantly (at least a 2-gene of difference between clusters) are indicated in bold. Percentages in parentheses indicate the rounded percentage of genes from each category found in a cluster. Genes whose function could not be associated with a category were annotated as Unclassified. Genes without a predicted function were annotated as Unknown.

CAZyme genes were more represented in clusters 8 and 9. These clusters group the most induced genes during induction for both strains and gather most of the genes encoding enzymes linked to cellulose degradation. We found among them the main endoglucanase genes *cel7b*, *cel5a*, *cel12a*, *cel45a* and *cel74a* (Trire2:122081, Trire2:120312, Trire2:123232, Trire2:49976, Trire2:49081, respectively), cellobiohydrolase *cel6a* (Trire2:72567) and swollenin *swo1* (Trire2:123992). The main cellobiohydrolase, *cel7b*, is absent from our dataset as induction is so strong that a saturating signal was obtained under all conditions. Also noteworthy is the presence of the lytic polysaccharide monooxygenase *cel61a* (now in the CAZy AA9 family) (Trire2: 73643) [35]. These clusters also include 12 other genes coding for glycoside hydrolases (GH) either already characterized as ß-galactosidase *bga1* (Trire2:80240) [36], ß-glucosidases *bgl2*, *cel1b, cel3c* (Trire2:120749, Trire2:22197, Trire2:82227, respectively) or putative ones [23,37,38].

Genes related to secretion are found predominantly in cluster 5 (only induced in RUT C30), and most of them belong to the ERV and SEC families. Other well-known genes that belong to the secretion system, *bip1*, *pdi1*, *cne1* and *sar1* (Trire2:122920, Trire2:122415, Trire2:73678, Trire2:61408, respectively), were identified in clusters 5 and 8. Concerning genes encoding transcriptional regulators, most of them belong to cluster 5. They include the xylanase regulator *xyr1* (Trire2:122208) and two genes (Trire2:77513 and Trire2:122523) whose overexpression was shown to increase the cellulase activity at least 1.5-fold with lactose as inducer [17]. Gene Trire2:77513 was named *ace3* by the authors because of its importance in the cellulase induction and in the activation of other cellulase genes. Outside cluster 5, a putative xylanase repressor *xpp1* (Trire2:122879) and a gene involved in carbon catabolite repression and ubiquitination *creD* (Trire2:81690) [39] were found in cluster 1.

Transporter genes were identified predominantly in cluster 7, only induced in NG 14. However, those described as sugar transporters [24] are mainly found in cluster 8. Genes from primary metabolism and energy metabolism were found mostly in cluster 4. Genes from this category included the down-regulated gene aldose epimerase *aep1* (Trire2:22415), which is involved in the catabolism of galactose via the Leloir pathway. In contrast, the D-xylose reductase *xyl1* (Trire2:107776) from the alternative galactose oxidoreductive pathway was found in the up-regulated cluster 5 (specific to RUT C30). Genes from the categories secondary metabolism, signal transduction, detoxification and stress response were also present and distributed among several clusters.

Except for cluster 9, no cluster was specific to a single category. However, clusters can be characterized by two or three main functional categories, suggesting a good match between functional categories and expression profiles, except for clusters 3 and 6, which could not be related to a specific functional category. Cluster 3 shares genes from the categories of clusters 2 and 4; therefore, it is close to them both in terms of functional categories as well as in terms of expression profile. Cluster 6 contains genes probably related to cellulase production, as it was the only down-regulated cluster specific to RUT C30 during protein production.

### Few genes have a higher basal expression in RUT C30

**Figure 4:**
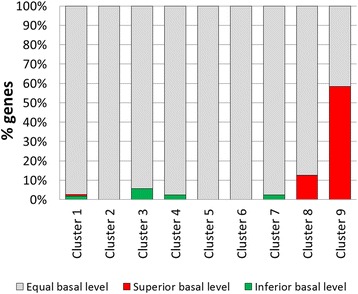
**Differences in gene expression before induction of protein production (T0) in NG 14 and RUT C30 identified by RNA-seq.** The percentage of genes with superior basal level (red bars), inferior basal level (green bars) or equal basal level (grey) is shown for each cluster.

As we were surprised by the large number of genes regulated in the NG 14 strain, we attempted to explain the apparent loss of up- and down-regulation in the RUT C30 strains. To achieve this, the time 0 references from RUT C30 and NG 14 were compared using RNA-seq experiments (Additional file 6: Table S3). From the 568 differentially expressed genes found using microarrays, we identified by RNA-seq only 23 whose expression was different between the two strains (Table 2), meaning that 95.9% of the differentially expressed genes during the protein production have the same basal level in these strains. The distribution of these genes between each cluster shows that most of them are included in clusters 8 and 9 (Figure 4). Most of these genes are cellulase genes that have a higher basal expression in RUT C30 and that represent 60% of cluster 9 (seven genes) and 10% of cluster 8 (four genes). This may explain the apparent lower induction of these genes in RUT C30 and is compatible with our previously published results positively correlating induction ratios and protein production [34]. However, the small number of genes induced or repressed prior to the lactose induction suggests that expression patterns observed between the two strains indeed reflect different behaviours regarding the lactose induction signal.

**Table 2:** **Genes regulated after lactose induction with different basal expression between NG 14 and RUT C30**

**Transcript ID**	**Cluster**	**Annotation**	**Ratio RUT C30/NG 14**	**Average reads**
59272	1	Putative MFS transporter	-4.78	17462
82032	1	Putative protein of unknown function	4.46	32445
121491	1	Putative glycosyltransferase family 4	4.21	38826
61374	3	Putative MFS transporter	-5.99	7537
74156	3	Putative secreted pepsin PROA	-4.92	7152
3717	4	Putative 2-keto-3-deoxy-L-galactonate aldolase	-4.86	7427
76155	4	Putative acid phosphatase	-4.85	23883
122582	4	Putative mitochondrial dihydrodipicolinate synthase	-4.38	8170
69115	7	Putative dienelactone hydrolase	-5.11	27147
69944	8	Putative glycoside hydrolase family 31	7.01	1685
73643	8	Glycoside hydrolase family 61 EGL4/CEL61A	4.34	4641
121127	8	Glycoside hydrolase family 3 BXL1	5.65	6623
123967	8	Hydrophobin HFB3	6.8	2548
69276	9	Putative glycoside hydrolase family 30	4.15	556
72567	9	Glycoside hydrolase family 6 CBH2/CEL6A	4.64	10892
73638	9	Secreted cellulose induced protein CIP1	6.37	1326
76210	9	Glycoside hydrolase family 62 ABF2	7.65	653
120312	9	Glycoside hydrolase family 5 EGL2/CEL5A	7.41	2331
120961	9	Glycoside hydrolase family 61 CEL61B	4.36	434
121418	9	Carbohydrate esterase family AES1	6.76	1027
60945	Not clustered	Putative MFS transporter	-4.51	6194
79816	Not clustered	Putative transcriptional regulator GAL80	-9.6	59020
123251	Not clustered	Putative NADH-quinone oxidoreductase	4.67	16873

Samples from T0 (before lactose induction) from NG 14 and RUT C30 were compared by means of RNA-seq experiments. The relative transcript abundance was measured in reads per kilobase of exon per million mapped sequence reads (RPKM). The log2 ratio of the RPKM values between RUT C30 and NG 14 were used to identify differentially expressed genes.

### Mutations in NG 14 and RUT C30 marginally affected regulated genes during lactose induction

In order to establish whether the differential expression of some genes during production was linked to the mutations that had been accumulated during mutagenesis of QM6a to NG 14 and subsequently RUT C30, we compared the gene expression profiles obtained in this work with the mutations found in NG 14 and RUT C30 (Additional file 7: Table S4 [21,22,25,37]). We systematically scanned for genes in an 800-bp window around each mutation. From the list of 165 mutations considered physically close enough to a gene to have a phenotypic effect, only 5 involved differentially expressed genes in at least one strain. Four mutations were specific to the mutagenesis towards RUT C30, which accounted for a total of 76 mutations. Interestingly, all four mutations relate to genes clustered in cluster 1 and cluster 5 (Table 3).

**Table 3:** **Inventory of the mutations affecting genes regulated during induction by lactose**

**Transcript ID**	**Cluster**	**Annotation**	**Strain**	**Mutation**	**Position**	**Reference**
**NG 14 and RUT C30 strains**						
1751	1	Putative FAD monooxygenase	RUT C30	SNV	Promoter	[21]
54511	5	Putative POZ domain protein	RUT C30	SNV	Promoter	[21]
56077	5	Putative transcription factor	RUT C30	SNV	Promoter	[21]
77513	5	Putative fungal C6 transcription factor	RUT C30	SNV	Exon	[21]
121087	Not clustered	Putative 4Fe-4S ferredoxin domain protein	NG 14 & RUT C30	SNV	Promoter	[21]
**Other cellulase improved strains**						
54736	1	Putative ATP-dependent RNA helicase DED1	KDG-12 PC-3-7	SNV	Promoter	[23]
58427	1	Putative ATP-dependent RNA helicase DBP2	PC-3-7	SNV	Exon	[24]
71380	1	Putative 3-hydroxy-3-methylglutaryl-coenzyme A reductase	PC-3-7	SNV	Exon	[24]
78836	1	Putative mitochondrial phosphate carrier	PC-3-7	SNV	Exon	[24]
82512	1	Putative 26S proteasome transcription factor RPN4	PC-3-7	SNV	Promoter	[24]
122036	1	Putative 40S ribosomal protein S2 RPS2	QM9414 KDG-12 PC-3-7	InDel & SNV	Promoter & Terminator	[25]
63882	4	Putative mitochondrial 3-hydroxyisobutyryl-CoA hydrolase	PC-3-7	SNV	Terminator	[24]
124031	4	Putative 20S proteasome alpha 3 subunit PRE9	PC-3-7	SNV	Promoter	[24]
6108	5	Putative SAM binding domain protein	QM9414 KDG-12 PC-3-7	InDel	Intron	Unpublished data
112390	5	Putative WD40 repeat domain protein	QM9414 KDG-12 PC-3-7	InDel	Terminator	Unpublished data
120688	5	Putative protein of unknown function	QM9414 KDG-12 PC-3-7	InDel	Exon	Unpublished data
110853	6	Putative glutathione S-transferase	PC-3-7	SNV	Terminator	[24]
77481	8	Putative D-xylulose 5-phosphate/D-fructose 6-phosphate phosphoketolase	PC-3-7	SNV	Exon	[24]
120749	8	Glycoside hydrolase family 1 BGL2	KDG-12 PC-3-7	SNV	Exon	[23]

We differentiate common mutations to RUT C30 and NG 14 strains from the mutations specific for RUT C30 strain. Mutations in regulated genes described in other cellulase improved strains are shown. Mutations are described as single nucleotide variation (SNV) and deletions or insertions (InDel). Affected gene region is also provided.

Because of the low correlation between genes that were mutated in the RUT C30 lineage and their expression data, we extended our comparison to mutations found in other cellulase improved strains [23-25] (Table 3). We found 14 mutations affecting differentially expressed genes. Most of them were again found in cluster 1 (six genes) and in cluster 5 (three genes), which is considerably higher than those in the NG 14 RUT C30 lineage.

## Discussion

### A refined view of protein production by *T. reesei* under an industrial regime

A number of transcriptome studies have already been published on *T. reesei* cellulase production. However, most of them have been performed with the low producer strain QM9414 [26-28]. In addition, most studies have been conducted in batch mode, preventing separation between growth and enzyme production and making carbon source availability difficult to control [26,37]. In addition, lactose, one of the preferred soluble substrates for industrial cellulase production, has received only limited interest [26,27]. One study was conducted with strain RUT C30 in chemostat cultures and focussed on the physiology of protein production [6]. These studies are a significant contribution to the fundamentals of cellulase production by *T. reesei*. However, the events that take place under industrial conditions (lactose as carbon source, near-zero residual carbon source, near-zero growth, constant pH) have not been studied yet. This study presents for the first time a transcriptome analysis of early induction by lactose in *T. reesei* under a feeding regime mimicking an industrial protocol [19]. The use of these controlled conditions may also explain the detection of a smaller number of genes responding to the lactose induction (below 600, all strains and time points considered) compared to previous studies that report more than 1,000 regulated genes [26-28].

Despite quantitative differences in the number of genes involved, our data are in agreement with previously published studies, and the main actors of enzyme production are found. Several genes encoding proteins belonging to the secretion system (*bip1, pdi1, cne1, sar1*), several chaperones as well as SEC and ERV family proteins account both for an induction of the secretion system and an enhancement of protein quality control (ERAD and UPR responses) [7-9]. Yet fewer CAZymes have been found in our study than in previous studies, but those found are the main components of the secretome. For example, we retrieved most CAZyme genes (19 of 26) from our results, including all major cellulases, in a list of 63 CAZymes from a transcriptome analysis of the strain QM9414 grown in a lactose medium [26]. These differences might be due to the strain and culture conditions and the fact that our study focussed on early induction. It is worth noticing that a previous work on RUT C30 two-dimensional electrophoresis already reported a secretome focussed on the main enzymes [19], which is compatible with our results. However, the use of different experimental designs or other analysis methods such as deep sequencing might also be partly responsible.

Relatively few transcription factors are induced or repressed, but while the main regulator *xyr1* is strongly induced, other known components of the cellulolytic enzymes regulon (*ace1*, *ace2)* have not been detected [4,34]. This could be due to the fact that transcriptome analysis has a limited sensitivity or that activation of these factors is mainly post-translational. It is noteworthy that the recently described *ace3* transcription factor is strongly induced [17]. Interestingly, the aldose epimerase gene 1 *aep1* is repressed in accordance with previous studies that show that the ß-D-galactose originating from lactose is catabolized via the reductive galactose catabolic pathway and does not require mutarotation to α-D-galactose, which is a prerequisite for catabolism via the Leloir pathway [40]. This coincides also with the observation that the gene encoding enzyme of the first step of the alternative galactose pathway *xyl1* is strongly induced. We also confirm the induction of genes encoding putative lactose transporters as recently reported [24,41]. Among them, the *crt1* gene (Trire2:3405) has been recently described as having a pivotal role in the lactose induction of cellulase genes, either as a lactose transporter or a cellulose sensor [24,26,42]. Three other transporters were highly up-regulated during growth on cellulose (Trire2:79202, Trire2:56684 and Trire2:54632). Several intracellular β-glucosidases were also regulated [23].

The relatively small number of detected genes and the fact that the majority of genes already being reported as having an impact on cellulase production are present in our dataset prompt us to believe that the data presented here is a very specific picture of cellulolytic enzymes induction by lactose. This is especially true for the RUT C30 strain that does not grow at all during the process. This study also presents a real-life picture of genes involved in enzyme production under an industrial carbon feeding regime, in other words, those which most probably have an impact on process performances.

### Comparison of NG 14 and RUT C30: a kinetic view of enzyme production

We used a cellulase production protocol originally adapted to a RUT C30 derivative, the strain CL847 [29,43]. This protocol mainly uses tuning of lactose feeding to maintain the strain under carbon limitation and thus ensures high cellulase production. We have previously shown [34] that this protocol could also be used for RUT C30 and may lead to some protein production in the lower producer strain QM9414 too, though with a more than 10-h lag phase and some carbon source accumulation before the start of production. The mutant strain NG 14 behaved differently, displaying a much reduced lag phase before onset of production, but - most importantly - was able to take up lactose as efficiently as RUT C30, so that the growth of both strains was carbon limited. This allowed us to investigate how the two strains reacted to the same inducing carbon flux.

**Figure 5:**
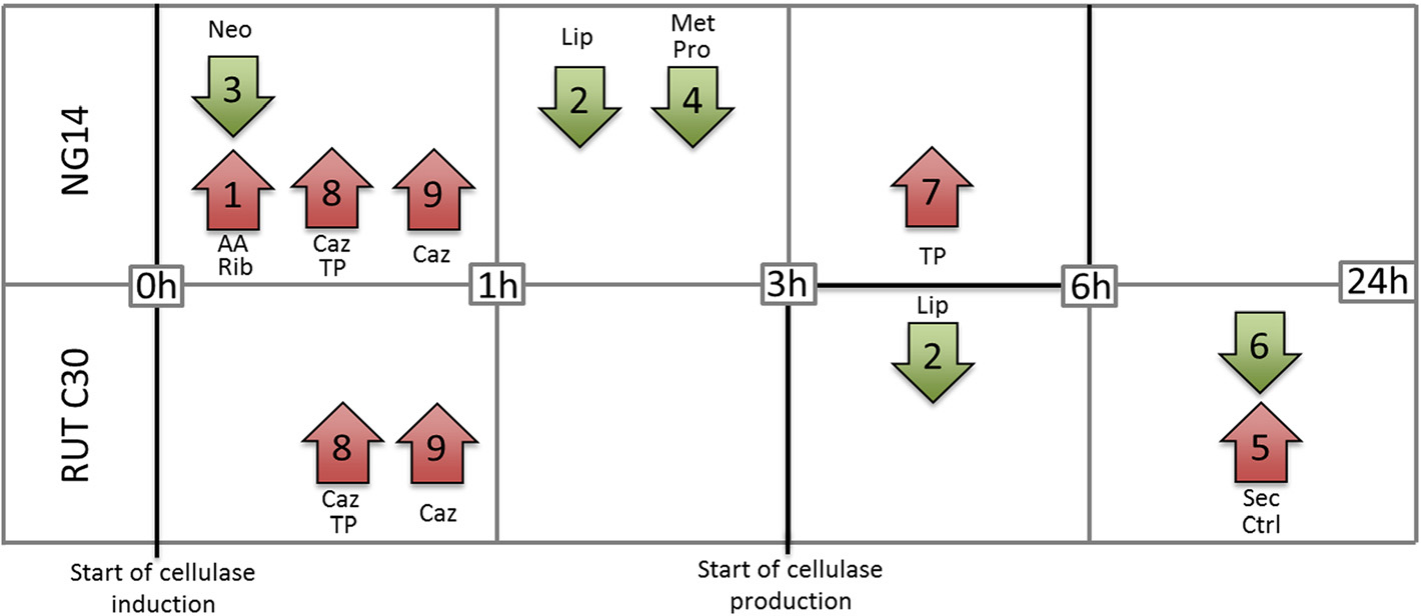
**Schematic view of gene expression kinetics in NG 14 and RUT C30.** Approximate regulation timing for each cluster is shown, and the number of each cluster is indicated inside each arrow. Red up-arrows and green down-arrows represent up- and down-regulated clusters respectively. Black bold line indicates the start time of protein induction and production in each strain. Main functional categories found in each cluster are indicated as Neo (gluconeogenesis), Rib (ribosome biogenesis), AA (amino acid metabolism), Lip (lipid metabolism), Caz (CAZymes), TP (transporter), Met (metabolism), Pro (extracellular protein degradation), Sec (secretion) and Ctrl (transcriptional control).

NG 14 and RUT C30 have seldom been compared for their respective performances [3,44,45]. Our data showed that RUT C30 has a specific productivity twice as high as that of NG 14 during the first 50 h of the fed-batch phase. Moreover, two main differences are reflected by the production curves (Figure 1); RUT C30 starts production at least 3 h earlier, and steady-state productivity during fed-batch is higher than for NG 14. A comparison of differentially expressed clusters found for each strain and sorted by time point is shown in Figure 5. It is interesting to note that no cluster is temporally correlated with the onset of protein production.

We investigated if the differences in gene regulation found in RUT C30 could be explained by the action of the carbon catabolite repressor CRE1 (which is truncated in RUT C30, [11]) because it would regulate some genes in NG 14 that are no longer regulated in RUT C30. Based on our previous work on the *cre1* regulon [9], we only found 66 genes in the present dataset to be potentially subject to carbon catabolite repression (data not shown). However, they were distributed in almost all the clusters, and have no common expression pattern with our previous study. While catabolite repression is probably operating in strain NG 14 (see below), the complex effect of CRE1 prevents us from formally identifying CRE1 “fingerprints” in our dataset. However, it is tempting to attribute the 3-h delayed production in NG 14 to the time needed to relief of catabolite repression.

Clusters 8 and 9, which group genes induced early in both strains (mainly cellulases and the *xyr1* regulator), do not explain by themselves the delay between NG 14 and RUT C30. However, genes from this cluster are strongly down-regulated in NG 14 before lactose induction (Figure 4). This observation can probably be attributed to the *cre1* truncation in RUT C30 [10,11,34], and explains the counter-intuitive observation that cellulases are more induced in NG 14 than in RUT C30. While the induction kinetics is the same, the higher absolute transcript levels in RUT C30 may be related to the earlier secretion of proteins in this strain, as previously suggested [34], and reflect a clear CRE1 signature. The recent observed effect of CRE1 on nucleosome positioning on the *cbh1* promoter suggests a broad role in chromatin remodelling for this regulator and thus alteration of the expression of many genes besides direct targets such as *xyr1* and cellulase genes [46]. The same work and another recently published one also present increasing evidences that the *cre1* truncation in RUT C30 is not equivalent to a full deletion [47].

Basal induction level comparisons between the two strains could not explain any other regulatory events that would be specific to NG 14, such as the early down-regulation of clusters 2 to 4, early up-regulation of cluster 1 and late up-regulation of cluster 7. Down-regulated clusters 2 to 4 comprise genes related to metabolism, proteasome, gluconeogenesis and lipid metabolism. Down-regulation of these genes in NG 14 could be an adaptation to lactose feeding and end of carbon starvation. Also, a large number of NG 14 up-regulated genes in cluster 1 (52) correspond to ribosome biogenesis. Note that ribosomal proteins were not identified in our previous study of *cre1*-deletants [9], and therefore this effect appears to be *cre1*-independent. However, without genetic engineering of NG 14 or RUT C30, we cannot formally rule out a direct or indirect CRE1 effect.

Clusters 5 and 6 group most of the genes that are specific to RUT C30. Interestingly, a significant induction of these clusters occurs after the onset of protein production in RUT C30. Some of these genes might be responsible for the higher productivity of this strain. Indeed, these clusters feature the highest percentage of regulators and secretion-related genes of our dataset. At these later time points, with catabolite repression relieved in both strains, it is possible to speculate that these differences are CRE1-independent. We have previously speculated on an effect caused by a relief from CRE1-carbon catabolite repression, and this can still not be ruled out by the present data, but this effect is still weakly documented and poorly understood [9,34].

In conclusion, we observed transcriptional differences between the *T. reesei* cellulase producer mutant strains NG 14 and RUT C30 both before (genes that might be involved in the start of production) and after (genes that might be involved in productivity phenotype) the start of protein production. While the majority of these differences may be attributable to the translation of a truncated CRE1 protein in strain RUT C30, the gene expression kinetic patterns observed suggest that other mutations may also have an effect on the strains’ performances and/or transcriptomes. For instance, the *bglr* Zn_2_C_6_ transcription factor, mutated in RUT C30 and in a strain from an independent lineage PC-3-7 but not in NG 14, was demonstrated to have an effect on cellulase production [21,23]. Conversely, the study of clusters 8 and 9 points out a role for carbon catabolite relief independently from lactose induction. The higher cellulase transcript levels might play a role in the earlier set of secretion in RUT C30.

### The cellulase induction system was remotely altered by the NG 14 to RUT C30 mutagenesis

In our previous work [21], complemented with new sequencing projects, we have been able to identify a nearly complete list of genes impacted by mutations of NG 14 and RUT C30: 130 by single nucleotide substitutions, 3 by small insertions or deletions and 32 by large structural variations. We compared these data with transcriptome data obtained in this work. Surprisingly, only about 3% of genes affected by mutations (either in promoter, terminator or coding sequence) are differentially expressed during the early phase of induction by lactose. This observation can be explained by the fact that many mutations may or may not modify gene functions but do not affect their expression. This is true in particular for transcription factors, whose action is not necessarily regulated by transcription. Conversely, a gene can be affected by a mutated transcription factor without being mutated itself. Nevertheless, our previous study already identified several genes involved in cellular processes such as secretion or protein maturation. It is striking to see that genes involved in the same processes are induced during induction of protein production and yet none of the mutated genes displayed a modified level of expression. Only the *ace3* gene (Trire2:77513) is a notable exception: it is at the same time induced in our datasets (and in other teams’ [17]), mutated in RUT C30, and has been identified as having an effect on cellulase production. The mutation in RUT C30 is a single nucleotide variation leading to a premature stop codon instead of a glutamine amino acid. This truncated C-terminus region is probably of regulatory importance, as in other Zn_2_Cys_6_ transcription factors [12]. This could lead to a relief from a potential repression, mimicking the effect of its overexpression [17]. It is interesting to note that other mutations affect promoters, and that three of them concern genes in cluster 5 that are specific to RUT C30 and possibly linked to productivity.

Intriguingly, a larger number of genes affected by mutations found in the independent lineages QM9414, KGD-12 and PC-3-7 were found in our dataset as well, and they are mostly located in clusters 1 and 5 [23-25]. This strengthens the importance of these clusters, particularly of cluster 1. It is possible that other uncharacterized genes in these clusters could be attractive targets for further genetic engineering of the strains.

## Conclusions

This study describes for the first time whole-genome transcriptional events for cellulose-producing strains of *T- reesei* in a protocol resembling industrial feeding conditions. While confirming previous observations, more than 500 genes have also been identified as involved in the process. Some of them might be targeted for further improvement of strains. This assumption is strengthened by our observation that the identified induction system is largely untouched in the NG 14-RUT C30 lineage by previous mutagenesis experiments, potentially leaving room for optimization, which was not guaranteed given the extensive work that was performed on these strains [48].

Random mutagenesis is and will remain a choice method for strain improvement, especially for improving complex phenotypes or poorly characterized organisms. Our data show that a combination of these conventional approaches together with genome and transcriptome analysis can help to sort out mutations, improving strain performances, and provide further targets for genetic engineering.

## Materials and methods

### Fungal strains and culture conditions

NG 14 (ATCC 56767) and RUT C30 (ATCC 56765) *Trichoderma reesei* strains were used in this study. Cultivations in the bioreactor were performed as previously described [34]. Frozen spores were used to inoculate a Fernbach flask containing 250 mL of the previously described culture medium [19]. Cultivation was carried out at 30°C with stirring at 150 rpm. After 72 h, the medium containing mycelia was used as an inoculum for bioreactor culture. The cellulase production was performed in a 4-L bioreactor under a two-phase cultivation procedure: strains were first grown in 2 L of medium containing 30 g.L^-1^ glucose as carbon source, at 28°C and pH regulated at 4.8 with 5.5 M NH_3_. The air flow was adjusted at 0.5 VVM and initial stirring was set at 400 rpm. This parameter was gradually increased to maintain pO_2_ above 40% oxygen saturation. When initial glucose was close to depletion (<0.5 g L^-1^ of initial glucose content), the fed-batch phase was initiated. During this second phase, a 250 g L^-1^ lactose solution was injected at a 0.98 g h^-1^ rate. Samples were collected periodically to determine the biomass, carbon and protein concentrations.

### Analytical methods

Biomass concentrations were determined by gravimetric analysis. 10 mL of the collected culture medium containing mycelia was filtrated on 1.2-μL GF/C glass microfiber membranes. The biomass dry weight was measured 24 h after incubation of the membrane at 105°C. Measures were done in triplicate with a standard error of less than 10%. Glucose concentration during the batch phase was assessed by enzymatic reaction using an Analox Glucose analyser GM10 (Imlab) to predict the convenient time to start fed-batch. All carbon source concentrations were *a posteriori* quantified again by HPLC using an HPX-87P column (Bio-Rad) maintained at 85°C. He-degassed distilled water was used as an eluent at a flow rate of 0.4 mL L^-1^. Measures were done in triplicate with a standard error of less than 5%. Extracellular protein concentration was measured against a BSA standard (0 to 1.5 g L^-1^ range with second-order regression) by the Bradford method [31] using the Quick Start Bradford Protein Assay kit (Bio-Rad) or by the Lowry method [33] using DC Protein Assay (Bio-Rad). Measures were done in triplicate with a standard error of less than 5%. The overall cellulase activity of the samples was measured as filter paper (FP) activities using the IUPAC-recommended procedure [49].

### RNA sample preparation

RNA samples were prepared from mycelia powder obtained by grinding the filtrated biomass in liquid nitrogen. The powder was subjected to a phenol treatment using TRI Reagent Solution (Applied Biosystems). The extracted total RNA was then isolated with bromochloropropane, precipitated with isopropanol, washed with ethanol and solubilized in nuclease-free water following the manufacturer’s instructions. Samples were cleaned up following the Qiagen RNeasy procedure and subjected to an on-column DNase digestion with the RNase-Free DNase Set (Qiagen).

### Microarray hybridizations

The microarray data and related protocols are available at the GEO website (http://www.ncbi.nlm.nih.gov/geo/), under the study number [GSE60908]. Briefly, the RNAs from each experiment were reverse-transcribed and labelled with Cy3 or Cy5 dyes using an indirect labelling procedure and dye-switch on the two biological replicates. Hybridization and scanner procedures for the *T. reesei* DNA chip manufactured by Agilent and designed using the Teolenn software have been described previously [9,50].The data were normalized without background subtraction by the global lowess method performed with the Goulphar software [51]. The background threshold was calculated by adding two standard deviations to the average intensity of all the “not found” features. For each probe, the log2 hybridization ratio was linked to genome annotation from the Joint Genome Institute (JGI) website (http://genome.jgi-psf.org/Trire2/Trire2.home.html). The final log2 ratio for each transcript was obtained by averaging the “detectable” hybridization values from all probes located inside the coding sequence on the matching strand. Transcripts with no probe marked as “detectable” were discarded from further analysis. For the two biological replicates on each of the four experiments, we applied to the pretreated results the linear modelling approach implemented by lmFit and the empirical Bayes statistics implemented by eBayes from the LIMMA R package [52]. We selected the list of statistically regulated genes using a 5% significance threshold. Finally, we kept as the most highly regulated targets only transcripts with a final log2 hybridization ratio greater than 1 or less than -1.

### RNA-seq library preparation and analysis

Messenger (polyA+) RNAs were purified from 2 μg of total RNA using oligo(dT). Libraries were prepared using the strand non-specific RNA-seq library preparation TruSeq RNA Sample Prep Kits v1 (Illumina). Libraries were multiplexed by 2 on one single flowcell lane and subjected to 50 bp paired-end read sequencing on a HiSeq 2000 device. A mean of 53 ± 12 million passing Illumina quality filter reads was obtained for each of the two samples’ RNA-seq data analysis.

We performed RNA-seq analysis using the Eoulsan pipeline [53]. Before mapping, poly N read tails were trimmed, reads ≤11 bases were removed, and reads with quality mean ≤12 were discarded. Reads were then aligned against the *T. reesei* genome (version 2 from the JGI website) using the Bowtie mapper (version 0.12.7) [54]. Alignments from reads matching more than once on the reference genome were removed using the Java version of SAMtools [55]. To compute gene expression, *T. reesei* genome annotation from JGI (version 2) was used. All overlapping regions between alignments and referenced exons were counted.

To analyse the gene expression level, the relative transcript abundance was measured in reads per kilobase of exon per million mapped sequence reads (RPKM) [56]. The log2 ratios of the RPKM values were used to identify differentially expressed genes. To keep only the most differentially expressed genes, a threshold of 4 for the log ratio with a reads number greater than 100 was chosen. The RNA-seq gene expression data and raw fastq files are available in (Additional file 6: Table S3).

### Cluster analysis of microarray results

An expression matrix was built from all the genes sorted as strongly regulated in at least one time point and in one strain. The GEPAS pipeline [57] was used to filter out genes with 30% missing values, leading to 568 selected genes. From these genes, a clustering analysis was performed using the MultiExperiment Viewer software [58]. A combination of unsupervised hierarchical clustering and K-means algorithms was used to sort genes into clusters. First, an ascending hierarchical clustering was done using the Euclidean distance metric and the average linkage method. Results were used to determine the optimal number K of clusters (K = 10). The K-means algorithm was applied using Euclidean distance on the 568 gene expression profiles. In order to improve the robustness of results, five independent runs of K-means with a random initialization were performed. Then, an aggregation method using a co-occurrence threshold of 60% was used to obtain the final clusters. 532 genes were classified in 9 clusters.

### Gene identification, functional prediction and classification

Regulated genes were first identified according to their ID number by reference to the *T. reesei* genome website (http://genome.jgi-psf.org/Trire2/Trire2.home.html). In the case of genes that were either poorly or not yet annotated, orthologous genes in other fungal taxa (mainly *Neurospora crassa*, *Saccharomyces cerevisiae* and *Aspergillus nidulans*) were searched using the FUNGIpath database (http://embg.igmors.u-psud.fr/fungipath/) and the function predicted by similarity. Genes without orthologs were annotated with their domains either from the *T. reesei* genome website or from a search on InterPro (http://www.ebi.ac.uk/interpro/). Identified proteins were categorized according to the Functional Catalogue (FunCat [59]) and they were manually curated to include only proteins with a clear function associated with a category. Proteins whose function could not be associated with a functional category were annotated as “unclassified”. Genes without a predicted function were annotated as “unknown”. Full expert annotation of our expression matrix is available in Additional file 5: Table S2.

### Mutations analysis

For genome versus transcriptome comparison, a gene was considered affected by a mutation when a mutation fell within the window delimited by 800 bp before the start codon and 800 bp after the stop codon. The RUT C30 and NG 14 mutations list (Additional file 7: Table S4) has been set up with mutations identified in Le Crom *et al.*, Koike *et al.* and Arvas *et al.* [21,22,37] and cleaned up by comparison with mutations in other lineages (QM9414 Arvas *et al.* [37] and QM9136 Lichius *et al.* [12]).

## Additional files

Additional file 1: Figure S1. Cultivation data for individual fermentations of NG 14 and RUT C30 strains. Additional file 2: Figure S2. Extracellular protein concentration of RUT C30 strain measured by Lowry assay. Additional file 3: Table S5. Analytical measurements during fermentation of NG 14 and RUT C30 strains. Concentration measurements of biomass, extracellular proteins, glucose, lactose and galactose are shown for each time point of fed-batch cultures (see Materials and methods for details). Additional file 4: Table S1. Differentially expressed genes found specific for NG 14 or RUT C30 strains. The expression values columns are expressed as log2 expression ratio compared to the reference time 0. Headers indicate the experimental condition studied. Additional file 5: Table S2. Differentially expressed genes annotation. Genes identified as differentially expressed in at least one time point of the global transcriptome analysis performed to study cellulase induction in NG 14 and RUT C30 strains. Full expert annotation and FunCat categories are indicated for each gene. The expression values columns are expressed as log2 expression ratio compared to the reference time 0. Headers indicate the experimental condition studied. Additional file 6: Table S3. Gene expression values for all transcripts from NG 14 and RUT C30 cultures at T0. Samples from T0 (before lactose induction) from NG 14 and RUT C30 were compared by RNA-seq. The relative transcript abundance was measured in reads per kilobase of exon per million mapped sequence reads (RPKM). The log2 ratio of the RPKM values between RUT C30 and NG 14 were used to identify differentially expressed genes. Additional file 7: Table S4. Updated list of mutations in NG 14 and RUT C30 strains. We took into account published work as indicated in the text. Only mutations close to genes are shown (see Materials and methods for details).

## Abbreviations

CAZyme: carbohydrate active enzyme
ERAD: endoplasmic reticulum-associated degradation
ERV: endoplasmic reticulum-derived transport vesicles
FunCat: Functional Catalogue
GH: glycoside hydrolase
SEC: secretory
UPR: unfolded protein response

## References

[1] Vinci VA, Byng G (1999). Strain Improvement by Nonrecombinant Methods. Volume 2.

[2] Kubicek CP, Mikus M, Schuster A, Schmoll M, Seiboth B (2009). Metabolic engineering strategies for the improvement of cellulase production by *Hypocrea jecorina*. Biotechnol Biofuels.

[3] Peterson R, Nevalainen H: ***Trichoderma reesei*****RUT-C30 - thirty years of strain improvement.** *Microbiology (Reading, England)* 2011:58–68 10.1099/mic.0.054031-0 21998163

[4] Stricker AR, Mach RL, De Graaff LH (2008). Regulation of transcription of cellulases- and hemicellulases-encoding genes in *Aspergillus niger* and *Hypocrea jecorina (Trichoderma reesei)*. Appl Microbiol Biotechnol.

[5] Stricker AR, Grosstessner-Hain K, Würleitner E, Mach RL (2006). Xyr1 (xylanase regulator 1) regulates both the hydrolytic enzyme system and D-xylose metabolism in *Hypocrea jecorina*. Eukaryot Cell.

[6] Mach-Aigner AR, Pucher ME, Steiger MG, Bauer GE, Preis SJ, Mach RL (2008). Transcriptional regulation of xyr1, encoding the main regulator of the xylanolytic and cellulolytic enzyme system in *Hypocrea jecorina*. Appl Environ Microbiol.

[7] Strauss J, Mach RL, Zeilinger S, Hartler G, Stöffler G, Wolschek M, Kubicek C (1995). Crel, the carbon catabolite repressor protein from *Trichoderma reesei*. FEBS Lett.

[8] Seidl V, Gamauf C, Druzhinina IS, Seiboth B, Hartl L, Kubicek CP (2008). The *Hypocrea jecorina (Trichoderma reesei)* hypercellulolytic mutant RUT C30 lacks a 85 kb (29 gene-encoding) region of the wild-type genome. BMC Genomics.

[9] Portnoy T, Margeot A, Linke R, Atanasova L, Fekete E, Sándor E, Hartl L, Karaffa L, Druzhinina IS, Seiboth B, Le Crom S, Kubicek CP (2011). The CRE1 carbon catabolite repressor of the fungus *Trichoderma reesei*: a master regulator of carbon assimilation. BMC Genomics.

[10] Nakari-Setala T, Paloheimo M, Kallio J, Vehmaanpera J, Penttila M, Saloheimo M (2009). Genetic modification of carbon catabolite repression in *Trichoderma reesei* for improved protein production. Appl Environ Microbiol.

[11] Ilmén M, Thrane C, Penttilä M (1996). The glucose repressor gene *cre1* of *Trichoderma*: isolation and expression of a full-length and a truncated mutant form. Mol Gen Genet.

[12] Lichius A, Seidl-Seiboth V, Seiboth B, Kubicek CP (2014). Nucleo-cytoplasmic shuttling dynamics of the transcriptional regulators XYR1 and CRE1 under conditions of cellulase and xylanase gene expression in *Trichoderma reesei*. Mol Microbiol.

[13] Saloheimo A, Aro N, Ilmén M, Penttilä M: **Isolation of the*****ace1*****gene encoding a Cys**_**2**_**-His**_**2**_**transcription factor involved in regulation of activity of the cellulase promoter*****cbh1*****of*****Trichoderma reesei*****.** *J Biol Chem* 2000, **275:**5817–5825. 10.1074/jbc.275.8.5817 10681571

[14] Aro N, Saloheimo A, Ilmén M, Penttilä M (2001). ACEII, a novel transcriptional activator involved in regulation of cellulase and xylanase genes of *Trichoderma reesei*. J Biol Chem.

[15] Seiboth B, Karimi RA, Phatale PA, Linke R, Hartl L, Sauer DG, Smith KM, Baker SE, Freitag M, Kubicek CP (2012). The putative protein methyltransferase LAE1 controls cellulase gene expression in *Trichoderma reesei*. Mol Microbiol.

[16] Denton JA, Kelly JM (2011). Disruption of *Trichoderma reesei cre2*, encoding an ubiquitin C-terminal hydrolase, results in increased cellulase activity. BMC Biotechnol.

[17] Häkkinen M, Valkonen MJ, Westerholm-Parvinen A, Aro N, Arvas M, Vitikainen M, Penttilä M, Saloheimo M, Pakula TM (2014). Screening of candidate regulators for cellulase and hemicellulase production in *Trichoderma reesei* and identification of a factor essential for cellulase production. Biotechnol Biofuels.

[18] Pakula TM, Salonen K, Uusitalo J, Penttilä M (2005). The effect of specific growth rate on protein synthesis and secretion in the filamentous fungus *Trichoderma reesei*. Microbiology.

[19] Herpoël-Gimbert I, Margeot A, Dolla A, Jan G, Mollé D, Lignon S, Mathis H, Sigoillot J-C, Monot F, Asther M (2008). Comparative secretome analyses of two *Trichoderma reesei* RUT-C30 and CL847 hypersecretory strains. Biotechnol Biofuels.

[20] Martinez D, Berka RM, Henrissat B, Saloheimo M, Arvas M, Baker SE, Chapman J, Chertkov O, Coutinho PM, Cullen D, Danchin EGJ, Grigoriev IV, Harris P, Jackson M, Kubicek CP, Han CS, Ho I, Larrondo LF, De Leon AL, Magnuson JK, Merino S, Misra M, Nelson B, Putnam N, Robbertse B, Salamov AA, Schmoll M, Terry A, Thayer N, Westerholm-Parvinen A (2008). Genome sequencing and analysis of the biomass-degrading fungus *Trichoderma reesei* (syn. *Hypocrea jecorina*). Nat Biotechnol.

[21] Le Crom S, Schackwitz W, Pennacchio L, Magnuson JK, Culley DE, Collett JR, Martin J, Druzhinina IS, Mathis H, Monot F, Seiboth B, Cherry B, Rey M, Berka R, Kubicek CP, Baker SE, Margeot A (2009). Tracking the roots of cellulase hyperproduction by the fungus *Trichoderma reesei* using massively parallel DNA sequencing. Proc Natl Acad Sci U S A.

[22] Koike H, Aerts A, LaButti K, Grigoriev IV, Baker SE (2013). Comparative genomics analysis of *Trichoderma reesei* strains. Ind Biotechnol.

[23] Nitta M, Furukawa T, Shida Y, Mori K, Kuhara S, Morikawa Y, Ogasawara W (2012). A new Zn(II)(2)Cys(6)-type transcription factor BglR regulates β-glucosidase expression in *Trichoderma reesei*. Fungal Genet Biol.

[24] Porciuncula JDO, Furukawa T, Shida Y, Mori K, Kuhara S, Morikawa Y, Ogasawara W (2013). Identification of major facilitator transporters involved in cellulase production during lactose culture of *Trichoderma reesei* PC-3-7. Biosci Biotechnol Biochem.

[25] Vitikainen M, Arvas M, Pakula T, Oja M, Penttilä M, Saloheimo M (2010). Array comparative genomic hybridization analysis of *Trichoderma reesei* strains with enhanced cellulase production properties. BMC Genomics.

[26] Ivanova C, Bååth JA, Seiboth B, Kubicek CP (2013). Systems analysis of lactose metabolism in *Trichoderma reesei* identifies a lactose permease that is essential for cellulase induction. PLoS One.

[27] Bischof R, Fourtis L, Limbeck A, Gamauf C, Seiboth B, Kubicek CP (2013). Comparative analysis of the *Trichoderma reesei* transcriptome during growth on the cellulase inducing substrates wheat straw and lactose. Biotechnol Biofuels.

[28] Dos Santos CL, Pedersoli WR, Antoniêto ACC, Steindorff AS, Silva-Rocha R, Martinez-Rossi NM, Rossi A, Brown NA, Goldman GH, Faça VM (2014). Comparative metabolism of cellulose, sophorose and glucose in *Trichoderma reesei* using high-throughput genomic and proteomic analyses. Biotechnol Biofuels.

[29] Jourdier E, Poughon L, Larroche C, Monot F, Ben Chaabane F (2012). A new stoichiometric miniaturization strategy for screening of industrial microbial strains: application to cellulase hyper-producing *Trichoderma reesei* strains. Microb Cell Fact.

[30] Jourdier E, Cohen C, Poughon L, Larroche C, Monot F, Ben Chaabane F (2013). Cellulase activity mapping of *Trichoderma reesei* cultivated in sugar mixtures under fed-batch conditions. Biotechnol Biofuels.

[31] Bradford MM (1976). A rapid and sensitive method for the quantitation of microgram quantities of protein utilizing the principle of protein-dye binding. Anal Biochem.

[32] Gusakov AV, Shulga TN, Chekushina AV, Sinitsyn AP (2013). Comparison of three protein assays for purified cellulases and hemicellulases from fungi. Open J Anal Chem Res.

[33] Lowry OH, Rosebrough NJ, Farr AL, Randall RJ (1951). Protein measurement with the Folin phenol reagent. J Biol Chem.

[34] Portnoy T, Margeot A, Seidl-Seiboth V, Le Crom S, Ben Chaabane F, Linke R, Seiboth B, Kubicek CP (2011). Differential regulation of the cellulase transcription factors XYR1, ACE2, and ACE1 in *Trichoderma reesei* strains producing high and low levels of cellulase. Eukaryot Cell.

[35] Hemsworth GR, Davies GJ, Walton PH (2013). Recent insights into copper-containing lytic polysaccharide mono-oxygenases. Curr Opin Struct Biol.

[36] Seiboth B, Hartl L, Salovuori N, Lanthaler K, Robson GD, Vehmaanperä J, Penttilä ME, Kubicek CP (2005). Role of the *bga1*-encoded extracellular β-galactosidase of *Hypocrea jecorina* in cellulase induction by lactose. Appl Environ Microbiol.

[37] Arvas M, Haiminen N, Smit B, Rautio J, Vitikainen M, Wiebe M, Martinez D, Chee C, Kunkel J, Sanchez C, Nelson MA, Pakula T, Saloheimo M, Penttilä M, Kivioja T (2010). Detecting novel genes with sparse arrays. Gene.

[38] Foreman PK, Brown D, Dankmeyer L, Dean R, Diener S, Dunn-Coleman NS, Goedegebuur F, Houfek TD, England GJ, Kelley AS, Meerman HJ, Mitchell T, Mitchinson C, Olivares HA, Teunissen PJM, Yao J, Ward M (2003). Transcriptional regulation of biomass-degrading enzymes in the filamentous fungus *Trichoderma reesei*. J Biol Chem.

[39] Boase NA, Kelly JM (2004). A role for *creD*, a carbon catabolite repression gene from *Aspergillus nidulans*, in ubiquitination. Mol Microbiol.

[40] Fekete E, Seiboth B, Kubicek CP, Szentirmai A, Karaffa L (2008). Lack of aldose 1-epimerase in *Hypocrea jecorina* (anamorph *Trichoderma reesei*): a key to cellulase gene expression on lactose. Proc Natl Acad Sci U S A.

[41] Ivanen DR, Rongjina NL, Shishlyannikov SM, Litviakova GI, Isaeva-Ivanova LS, Shabalin KA, Kulminskaya AA (2009). Novel precipitated fluorescent substrates for the screening of cellulolytic microorganisms. J Microbiol Methods.

[42] Zhang W, Kou Y, Xu J, Cao Y, Zhao G, Shao J, Wang H, Wang Z, Bao X, Chen G (2013). Two major facilitator superfamily sugar transporters from *Trichoderma reesei* and their roles in induction of cellulase biosynthesis. J Biol Chem.

[43] Warzywoda M, Larbre E, Pourqui J (1992). Production and characterization of cellulolytic enzymes from *Trichoderma reesei* grown on various carbon sources. Bioresour Technol.

[44] Montenecourt BS, Eveleigh DE (1977). Preparation of mutants of *Trichoderma reesei* with enhanced cellulase production. Appl Environ Microbiol.

[45] Eveleigh DE, Montenecourt BS (1979). Increasing yields of extracellular enzymes. Adv Appl Microbiol.

[46] Ries L, Belshaw NJ, Ilmén M, Penttilä ME, Alapuranen M, Archer DB (2014). The role of CRE1 in nucleosome positioning within the *cbh1* promoter and coding regions of *Trichoderma reesei*. Appl Microbiol Biotechnol.

[47] Mello-de-Sousa TM, Gorsche R, Rassinger A, Poças-Fonseca MJ, Mach RL, Mach-Aigner AR (2014). A truncated form of the carbon catabolite repressor 1 increases cellulase production in *Trichoderma reesei*. Biotechnol Biofuels.

[48] Kazi FK, Fortman JA, Anex RP, Hsu DD, Aden A, Dutta A, Kothandaraman G (2010). Techno-economic comparison of process technologies for biochemical ethanol production from corn stover. Fuel.

[49] Ghose TK (1987). Measurement of cellulase activities. Pure Appl Chem.

[50] Jourdren L, Duclos A, Brion C, Portnoy T (2010). Teolenn: an efficient and customizable workflow to design high-quality probes for microarray experiments. Nucleic Acids.

[51] Lemoine S, Combes F, Servant N, Le Crom S (2006). Goulphar: rapid access and expertise for standard two-color microarray normalization methods. BMC Bioinformatics.

[52] Smyth GK (2004). Linear models and empirical Bayes methods for assessing differential expression in microarray experiments. Stat Appl Genet Mol Biol.

[53] Jourdren L, Bernard M, Dillies M-A, Le Crom S (2012). Eoulsan: a cloud computing-based framework facilitating high throughput sequencing analyses. Bioinformatics.

[54] Langmead B, Trapnell C, Pop M, Salzberg SL (2009). Ultrafast and memory-efficient alignment of short DNA sequences to the human genome. Genome Biol.

[55] Li H, Handsaker B, Wysoker A, Fennell T, Ruan J, Homer N, Marth G, Abecasis G, Durbin R (2009). The sequence alignment/map format and SAMtools. Bioinformatics.

[56] Mortazavi A, Williams BA, McCue K, Schaeffer L, Wold B (2008). Mapping and quantifying mammalian transcriptomes by RNA-Seq. Nat Methods.

[57] Vaquerizas JM, Conde L, Yankilevich P, Cabezón A, Minguez P, Díaz-Uriarte R, Al-Shahrour F, Herrero J, Dopazo J (2005). GEPAS, an experiment-oriented pipeline for the analysis of microarray gene expression data. Nucleic Acids Res.

[58] Howe E, Holton K, Nair S, Schlauch D, Sinha R, Quackenbush J: **MeV: MultiExperiment Viewer.** In *Biomedical Informatics for Cancer Research.* Springer (US); 2010:267–277

[59] Ruepp A, Zollner A, Maier D, Albermann K, Hani J, Mokrejs M, Tetko I, Güldener U, Mannhaupt G, Münsterkötter M (2004). The FunCat, a functional annotation scheme for systematic classification of proteins from whole genomes. Nucleic Acids Res.

