# Supplementary figures and images for: Kinetic transcriptome analysis reveals an essentially intact induction system in a cellulase hyper-producer Trichoderma reesei strain
Source: Biotechnol Biofuels. 2014 Dec 12;7:173. doi: 10.1186/s13068-014-0173-z (PMC4279801; doi:10.1186/s13068-014-0173-z)

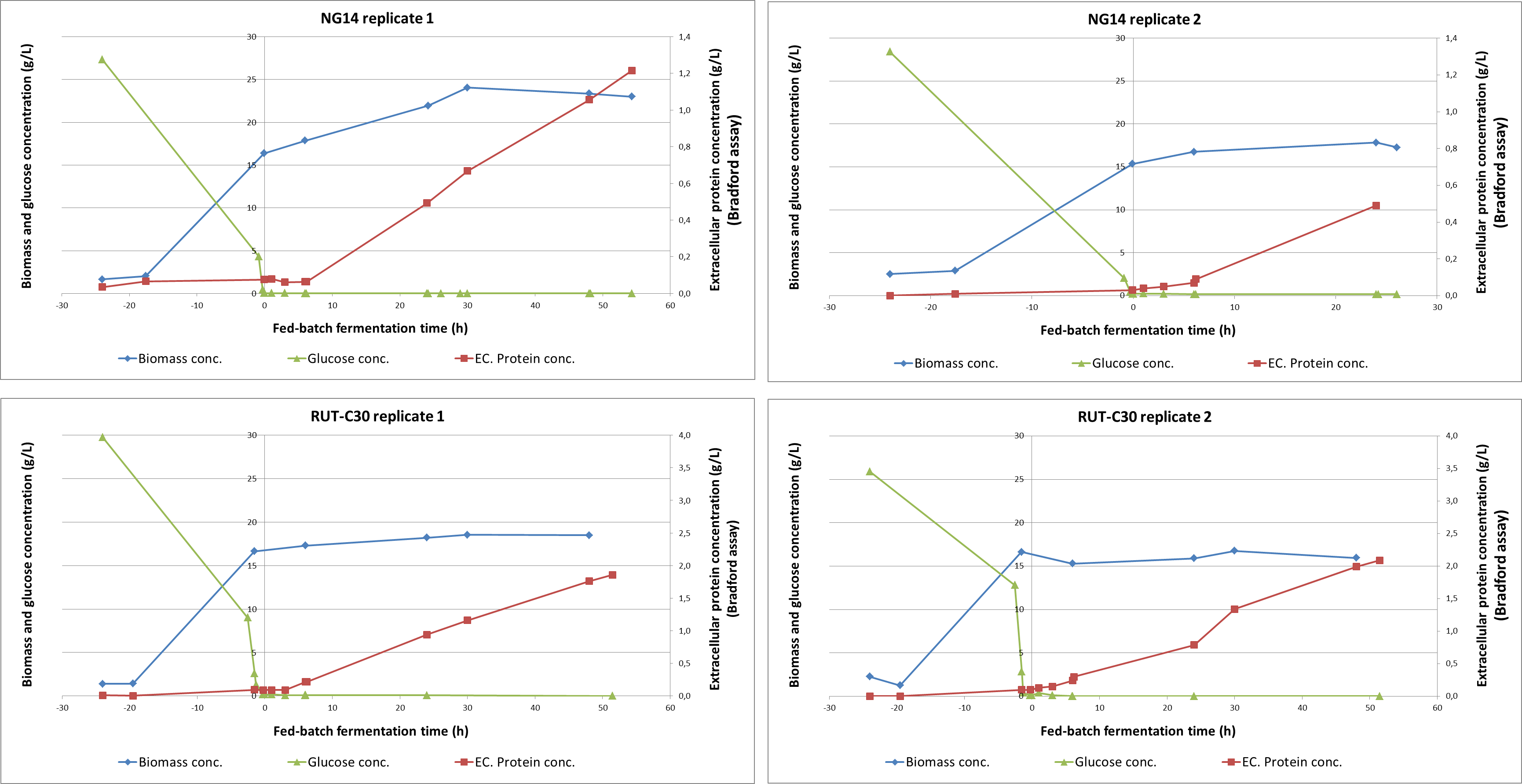

Supplement: Additional file 1: Figure S1. — Cultivation data for individual fermentations of NG 14 and RUT C30 strains. [file 13068_2014_173_MOESM1_ESM.png]

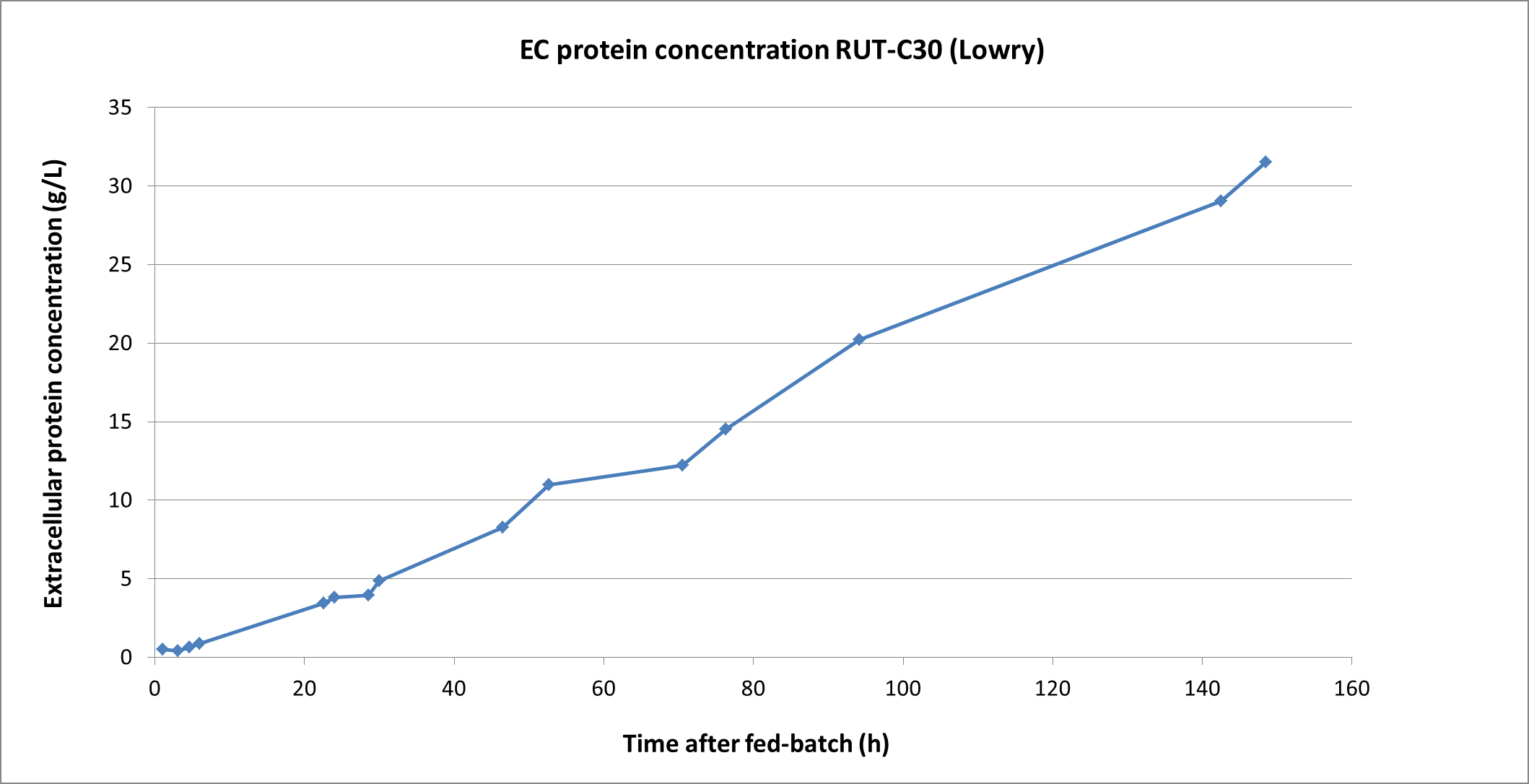

Supplement: Additional file 2: Figure S2. — Extracellular protein concentration of RUT C30 strain measured by Lowry assay. [file 13068_2014_173_MOESM2_ESM.png]
